# Pituitary Apoplexy: An Updated Review

**DOI:** 10.3390/jcm13092508

**Published:** 2024-04-24

**Authors:** Pedro Iglesias

**Affiliations:** Department of Endocrinology and Nutrition, Hospital Universitario Puerta de Hierro Majadahonda, Instituto de Investigación Sanitaria Puerta de Hierro Segovia de Arana, 28222 Madrid, Spain; piglo65@gmail.com

**Keywords:** pituitary apoplexy, hemorrhage, infarction, pituitary gland, hypopituitarism, conservative management, surgery

## Abstract

Pituitary apoplexy (PA) is an acute, life-threatening clinical syndrome caused by hemorrhage and/or infarction of the pituitary gland. It is clinically characterized by the sudden onset of headache. Depending on the severity, it may also be accompanied by nausea, vomiting, visual disturbances, varying degrees of adenohypophyseal hormone deficiency, and decreased level of consciousness. Corticotropic axis involvement may result in severe hypotension and contribute to impaired level of consciousness. Precipitating factors are present in up to 30% of cases. PA may occur at any age and sometimes develops during pregnancy or the immediate postpartum period. PA occurs more frequently in men aged 50–60, being rare in children and adolescents. It can develop in healthy pituitary glands or those affected by inflammation, infection, or tumor. The main cause of PA is usually spontaneous hemorrhage or infarction of a pituitary adenoma (pituitary neuroendocrine tumor, PitNET). It is a medical emergency requiring immediate attention and, in many cases, urgent surgical intervention and long-term follow-up. Although the majority of patients (70%) require surgery, about one-third can be treated conservatively, mainly by monitoring fluid and electrolyte levels and using intravenous glucocorticoids. There are scoring systems for PA with implications for management and therapeutic outcomes that can help guide therapeutic decisions. Management of PA requires proper evaluation and long-term follow-up by a multidisciplinary team with expertise in pituitary pathology. The aim of the review is to summarize and update the most relevant aspects of the epidemiology, etiopathogenesis, pathophysiology, clinical presentation and clinical forms, diagnosis, therapeutic strategies, and prognosis of PA.

## 1. Introduction

Pituitary apoplexy (PA) is an acute and potentially fatal clinical syndrome caused by hemorrhage and/or infarction of the pituitary gland. The first known fatal case of hemorrhage from a PitNET was reported by Bailey in 1898 [1]. However, it was not until 1950 that Brougham et al. introduced the term “pituitary apoplexy” and described the clinical and pathologic findings in a series of five patients who died suddenly and whose autopsies revealed hemorrhagic necrosis in PitNETs [2].

PA is a medical emergency requiring immediate attention and, in many cases, urgent surgical intervention. Clinically, it is characterized by the sudden onset of headache, sometimes associated with nausea, vomiting, visual disturbances, and decreased level of consciousness. In addition, the development of acute hypopituitarism may compromise the hormonal function of the anterior pituitary and, in the case of corticotropic axis involvement, may contribute to a progressive deterioration of the level of consciousness with severe hypotension.

Given the complexity and severity of the clinical picture, PA should be managed urgently by a multidisciplinary team composed of emergency physicians, neuroradiologists, neurosurgeons, endocrinologists, and neuro-ophthalmologists. Therapeutic modalities and timing of intervention are still under debate. Thus, the therapeutic approach to patients with PA tends to be empirically individualized, and clinical outcome is closely related to the skills and experience of the multidisciplinary team [3]. Although urgent surgery is required for patients with worsening neurological symptoms, the optimal treatment (conservative or surgical) that will provide the best results for the majority of patients has not yet been clearly established.

This review analyzes and updates the main aspects related to the epidemiology, etiopathogenesis and pathophysiology, clinical presentation, clinical forms, diagnosis, therapeutic strategies, and prognosis of PA.

## 2. Epidemiology

**Figure 1:**
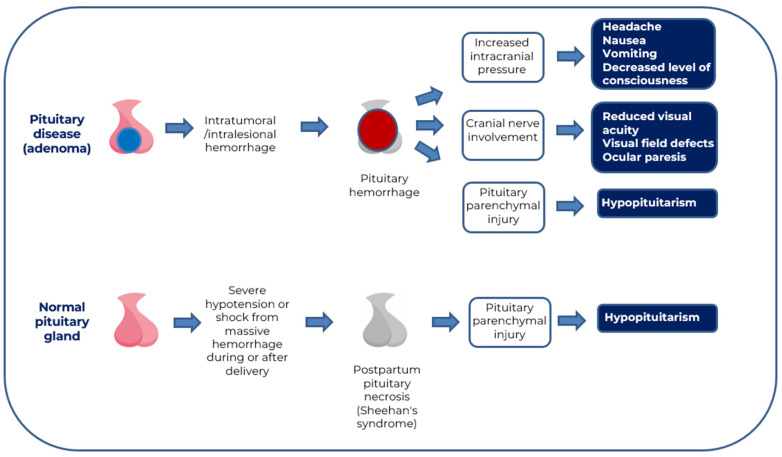
Mechanisms of pituitary injury and associated clinical symptoms in pituitary apoplexy.

PA is a very rare disease. Its incidence is around 0.17 episodes per 100,000 person-years [4] and its estimated prevalence is approximately 6.2 cases per 100,000 people [5]. It can develop in healthy pituitary glands or those affected by inflammation, infection, or tumor (Figure 1). PA occurs more frequently in men (67.5%, aged 50–60) than women, with a ratio of 2:1, being rare in children and adolescents [6,7,8,9,10]. PA can appear in any histologic pituitary tumor, however, it is more common in non-functioning PitNETs (NF-PitNETs) and prolactinomas [9,11,12]. Less frequently, PA has also been associated with other pituitary diseases such as Rathke’s cleft cysts [13], craniopharyngiomas [14], pituitary metastases, especially of renal cell carcinoma [15], pituitary tuberculosis [16,17], and hypophysitis [18,19].

The occurrence of PA in a previously healthy pituitary is usually due to severe hypotension or hypovolemic shock caused by massive hemorrhage during or after delivery resulting in ischemic necrosis of the pituitary gland (Sheehan’s syndrome) [20,21]. The incidence and prevalence of Sheehan’s syndrome in pregnant women varies according to different populations and geographic regions. Its incidence is low in developed countries due to the availability of adequate medical care during delivery and postpartum. However, in developing countries or countries with limited access to medical care, the incidence may be higher due to factors such as suboptimal delivery conditions and lack of adequate medical follow-up. In a population-based study from Iceland, Sheehan’s syndrome was diagnosed in 5.1 individuals per 100,000 population [22], whereas its prevalence among 11,700 women >20 years of age in northern India was approximately 3% [23]. In developed countries, Sheehan’s syndrome accounts for less than 6% of the causes of hypopituitarism [24,25], but in other countries, such as Pakistan and Turkey, this percentage is approximately 30% [26,27].

## 3. Pathophysiology

Several pathophysiological mechanisms have been implicated in the development of PA, including: (1) intralesional hemorrhage (PitNETs, infectious and inflammatory processes, and vascular malformations); (2) acute increase in pituitary blood flow (hypertensive crises, trauma, increased physical activity, and increased intracranial pressure); (3) increased blood flow demands in a growing PitNET whose needs exceed blood supply causing ischemic necrosis; (4) pituitary ischemia in situations of low blood supply, as in surgery, especially cardiac, radiotherapy, spinal anesthesia, and severe hypotension or hypovolemic shock after hemorrhage during childbirth; (5) pituitary and tumor hormonal stimulation (pituitary stimulation endocrine tests, especially TRH test, and the use of GnRH analogs, pregnancy, and exogenous estrogen therapy); and (6) coagulation disorders (thrombocytopenia, coagulopathies, and antiplatelet and anticoagulant drugs) [9,28,29,30,31].

There is evidence to support the idea that certain genetic conditions, as well as the action of certain cytokines and growth factors, may trigger pathways leading to pituitary necrosis or hemorrhage [32,33]. The poor vascularization of PitNETs plays a significant role in the activation of certain molecular mediators associated with tumor bleeding. However, the underlying molecular pathways are not fully understood and there are currently no molecular markers that predict the development of PA [30].

**Figure 2:**
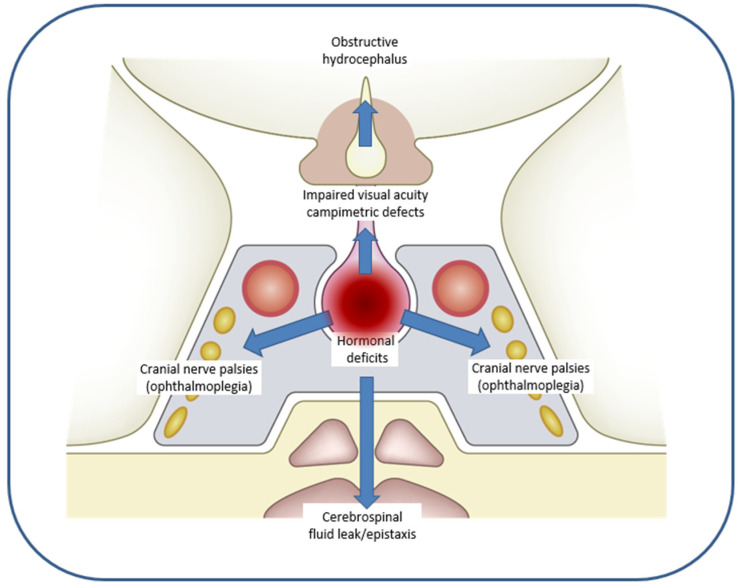
Compressive symptoms resulting from hemorrhage in pituitary apoplexy depending on adjacent structures involved.

Regardless of the underlying mechanism (hemorrhage or infarction), the extent of hemorrhage and/or necrosis causes an increase in intrasellar pressure, resulting in more or less pronounced compression of adjacent structures, which explains the diversity of clinical manifestations observed (Figure 1 and Figure 2) [31,34]. Superior compression on the optic chiasm is accompanied by alterations in visual acuity and campimetric defects, such as bitemporal hemianopsia, which may occur in up to 75% of patients. Lateral compression can affect the nerve structures of the cavernous sinus, causing oculomotor nerve palsy (ophthalmoplegia) in 50–70% of patients, most frequently affecting the third cranial nerve. Inferior compression may result in rupture of the sellar floor with formation of a cerebrospinal fluid (CSF) leak. Although hemorrhage is usually confined to the tumor, it may often be accompanied by extravasation of blood into the subarachnoid space, causing symptoms of meningeal irritation. In large macroadenomas with suprasellar extension, obstructive hydrocephalus may develop as a complication of PA (Figure 2) [7,35,36,37,38].

## 4. Precipitating Factors

Precipitating factors have been identified in up to 30–40% of PA cases [39]. These include arterial hypertension and diabetes mellitus; major surgery (especially coronary artery bypass graft surgery); antiplatelet, anticoagulant, and fibrinolytic therapy; coagulation disorders; pregnancy; complicated delivery; severe hypotension and shock; head trauma; pituitary stimulation tests with gonadotropin-releasing hormone (GnRH), thyrotropin-releasing hormone (TRH), corticotropin-releasing hormone (CRH), or insulin hypoglycemia; radiotherapy; GH- and ACTH-secreting PitNETs and large NF-PitNETs, especially silent corticotropinomas; aneurysm rupture; and medications such as estrogens, somatostatin analogs, and dopamine agonists [35,40,41,42,43,44,45,46,47,48,49,50]. In this context, it is important to inform all patients with pituitary tumors, especially those with potential precipitating factors, about the symptoms of PA for early detection and appropriate treatment.

## 5. Clinical Presentation and Natural History

The clinical presentation of PA depends on the speed of onset of infarction or hemorrhage and the volume of hemorrhage. These factors will influence the degree of compression of adjacent neuro-ophthalmologic structures and the pituitary gland. Therefore, the clinical presentation of PA may vary from patient to patient. It can be acute and severe (acute PA), with serious neurological deficits, coma, and even death. Alternatively, it may be subacute (subclinical PA), with milder symptoms developing over days or weeks [51,52,53].

Those cases of PA due to pituitary tumor infarction usually have less severe clinical symptoms at presentation, a longer course before presentation, and a better prognosis than those with hemorrhagic infarction or frank hemorrhage. However, hormone replacement needs are usually similar in both groups [53].

**Figure 3:**
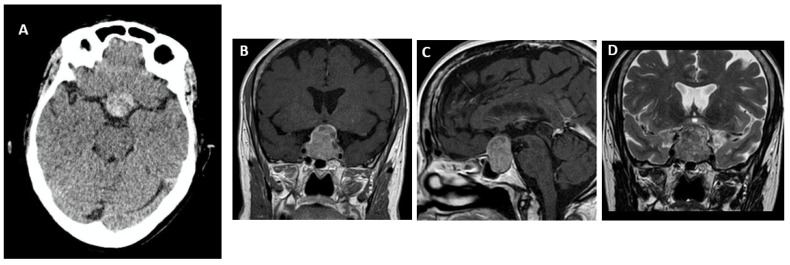
Imaging studies in a patient with acute pituitary apoplexy due to intratumoral bleeding. A 58-year-old woman with sudden onset of headache and visual loss in the postoperative period after aortic valve replacement. A CT scan of the brain (**A**) showed a sellar lesion (3 × 2.8 × 2 cm) with heterogeneous suprasellar extension with hyperdense content compatible with intralesional hemorrhage. MRI of the pituitary gland showed an expansive process involving the entire sella turcica with extension to the suprasellar cistern and the anterior region of the third ventricle. On paramagnetic contrast T1-weighted ((**B**), coronal; (**C**), sagittal) and T2-weighted ((**D**), coronal) sequences the signal is heterogeneous with a hyperintense area in the tumor dome and left posterolateral region.

PA should be suspected in sudden onset of severe retro-orbital, frontal, or suboccipital headache with nausea and vomiting and cranial nerve symptoms such as diplopia, palpebral ptosis, and mydriasis with variable level of consciousness (Figure 3). It may also be associated with decreased visual acuity with visual field involvement ranging from small field defects to complete bilateral vision loss. The development of partial or complete hypopituitarism is common [54,55].

The main symptoms associated with PA are headache (89%), ophthalmoplegia (78%) (mainly due to third cranial nerve palsy, followed by fourth and sixth cranial nerve palsies), vomiting (69%), campimetric defects (64%), and decreased visual acuity (52%) [56,57,58]. Hypopituitarism is frequent, with gonadotropin deficiency being the most common hormone deficiency (75%), followed by corticotropin deficiency (70%) and thyrotropin (50%) [59]. Almost all patients with PA have GH deficiency at diagnosis, although GH deficiency is not usually tested at the time of diagnosis [55,58]. Some authors have suggested that low serum prolactin levels may indicate a greater degree of pituitary destruction and, therefore, a lower likelihood of pituitary function recovery after surgery [31,36]. Hyponatremia may occur in up to 40% of patients with PA secondary to adrenal insufficiency, central hypothyroidism, or the syndrome of inappropriate secretion of antidiuretic hormone (SIADH) [52]. In rare cases, epileptic seizures, hemiplegia, and diabetes insipidus have also been reported [60,61,62].

Regardless of the type of treatment (conservative or surgical), the development of some degree of permanent hypopituitarism is common. Campimetric defects and visual acuity usually improve after treatment and ophthalmoplegia usually resolves in most cases. Recurrence of the underlying pituitary tumor and new episodes of PA are rare [36,54]. However, patients with functioning PitNETs require long-term follow-up due to the high risk of persistence/recurrence of endocrine hyperactivity [54].

## 6. Pituitary Apoplexy (PA) in Pituitary Neuroendocrine Tumors (PitNETs)

PA prevalence in different published series ranges from 0.6% to 10%, with an average of 2% of all surgically removed PitNETs [9,11]. The risk of developing PA in patients with NF-PitNETs and pituitary incidentalomas is between 0.2 and 0.6 events per 100 person-years [63,64]. Although PA has been classically associated with macroadenomas, it has also been reported, although with a lower prevalence (0.36%), in patients with pituitary microadenomas [5,9,11,12,64,65].

NF-PitNETs constitute 45% of all pituitary tumors that develop PA [56,66]. It has also been reported in non-functioning pituitary microadenomas, although less frequently than in macroadenomas [67,68].

It is well known that dopamine agonists used in the treatment of prolactinoma are a risk factor for PA. These drugs promote tumor regression due to reduction of lactotrope size and degenerative and necrotic changes in tumor cells. This association has been described mainly with bromocriptine, but there are also reported cases associated with the use of cabergoline, since it acts in the same way as bromocriptine by reducing the size of prolactinoma after inducing apoptosis of lactotrope cells [69,70,71]. Although dopamine agonists may precipitate PA, treatment can be continued after the PA to achieve hormonal and prolactinoma size control. PA has also been reported in pregnant women with micro-, macro-, and giant prolactinomas [43,72,73,74].

PA also develops in patients with acromegaly. In fact, the first described case of PA was in a patient with a somatotropinoma [1]. Sometimes the patient diagnosed with acromegaly may be complicated by fulminant PA [75]. The average age of affected patients is 37 years and there is no specific gender predilection. Patients usually present with invasive adenomas (Knosp grade ≥ 3). After PA, GH and IGF-1 levels tend to decrease, and spontaneous biochemical remission may occur along with partial or complete anterior or posterior (or both) pituitary insufficiency [76].

Although pituitary tumor apoplexy is the most commonly reported event (91%) in patients with spontaneous remission of Cushing’s disease [77], cases of tumor recurrence after PA have been described. Therefore, careful long-term follow-up is necessary [78].

### 6.1. Pituitary Apoplexy in Children and Adolescents

PA is a rare event in children and adolescents [8,79,80,81,82,83,84,85]. It has been reported in 1.8% to 7.5% of all surgically treated PitNETs in this population [86,87]. More recently, PA was found in 42.5% of pediatric and adolescent PitNETs [85]. In contrast to adult patients, in whom PA is more frequent in non-functioning PitNETs, in children and adolescents PA is more commonly seen in functioning PitNETs, mainly prolactinomas (73.5%) [83,85]. There is no sex predilection and the mean age at the time of diagnosis is 16.6 ± 2.6 years. Visual disturbances and hypopituitarism occur in approximately 40–45% of patients. No significant differences have been observed between PitNETs with and without PA in the degree of invasion according to the Knosp grading system and in the expression of Ki-67 proliferation index. Lastly, the tumor recurrence rate after surgery is 14.7% [85].

### 6.2. Pituitary Apoplexy in the Elderly

No large series of elderly patients with PA have been reported. Some articles have described experiences in isolated cases and small series [88,89,90,91]. With increasing life expectancy, the incidence of PitNETs in the elderly population is expected to increase, leading to an increased PA incidence. In addition, PA risk is increased by the frequent presence of comorbidities in the elderly that act as predisposing or precipitating factors, such as hypertension and diabetes, and the widespread use of antiplatelet and anticoagulant medications in this population. Given the high prevalence of prostate cancer in older men, it is important to remember that GnRH agonist androgen deprivation therapy for prostate cancer may precipitate PA in this population, especially in those with PitNETs [88].

PA in the elderly often presents with symptoms of hypopituitarism. In this population, non-specific symptoms of hypopituitarism may be attributed to other comorbidities, complicating clinical evaluation [89]. Although the presence of acute confusion in the elderly is usually due to delirium precipitated by infection, occasionally an episode of acute confusion may be the presenting form of PA, which should be considered especially if the patient has a history of PitNET [90].

For older patients with PA contraindicated for surgery, even with compression symptoms such as ophthalmoplegia, if conservative management has been effective and there has been no deterioration, the patient can be carefully monitored and conservative management can be continued [91].

### 6.3. Pituitary Apoplexy and Pregnancy

PA during pregnancy is a rare event. It is estimated to occur in about 1 in 10,000 term pregnancies, with a mean gestational age at diagnosis around 24 weeks of gestation, and approximately 10% of cases develop in the postnatal period (Sheehan’s syndrome). Often this event is the first clinical manifestation of a pituitary tumor, typically a macroadenoma, as these are more prone to bleeding [43,46,47,92,93].

During pregnancy, increased estrogen levels cause hypertrophy and hyperplasia of the lactotrope cells, resulting in an increase in pituitary volume [94,95]. In addition, pregnancy is known to be associated with an increase in the volume of prolactinomas, especially macroprolactinomas [96]. Finally, pregnancy is a prothrombotic state that may favor pituitary ischemic phenomena [97].

A recent review of 36 cases of PA during pregnancy showed that most cases occurred in the second trimester. At diagnosis, the mean age of the pregnant women was 29 ± 4 years and the mean gestational age was 25 ± 8 weeks. PitNETs were previously diagnosed in almost half of the patients (47%) (seven macroadenomas, four microadenomas, and six adenomas without specification of tumor size). Headache was the most common symptom at presentation (86%). Approximately 80% of pregnant women developed partial or complete hypopituitarism. The degree of hypopituitarism depends on the extent of the pituitary parenchyma involved. The most common hormonal deficiency was gonadotropins, followed by ACTH and TSH, and less commonly prolactin. Surgical management was required in more than half of the pregnant women (61%). Caesarean section was the mode of delivery in 15 cases, vaginal in six, and unknown in 48%. Maternal and fetal outcomes included three cases of preterm delivery and one maternal death [47].

## 7. Diagnosis

The clinical suspicion of PA is based on the acute symptoms presented by patients, characterized by the sudden onset of severe headache, usually retro-ocular and worsening with time; nausea and vomiting; visual disturbances (reduction of visual acuity, blurred vision, or reduction of visual fields); ophthalmoplegia due to involvement of oculomotor cranial nerves III, IV, and VI; and finally, in severe cases, a deterioration of the level of consciousness [98].

In order to make a diagnosis of PA, an imaging study is required. Magnetic resonance imaging (MRI) is the preferred option (sensitivity of approximately 90%) because it can visualize and detect hemorrhage, necrosis, and edema within the pituitary gland, the presence of a PitNET, and compression of the optic chiasm and other adjacent structures. It can also detect lesions that may mimic PA such as aneurysms, Rathke’s cleft cysts, craniopharyngioma, and mucocele [99]. In the acute phase (0–7 days), a hypointense signal is seen on T2-weighted images (T2WI) and an isointense or slightly hypointense signal is seen on T1-weighted images (T1WI). In the subacute phase (7–21 days), methemoglobin shortens the T1 relaxation time, causing the bleed to appear hyperintense on both T1WI and T2WI. In the chronic phase (>21 days), the presence of hemosiderin and ferritin causes marked hypointensity on both T1WI and T2WI [100,101]. Another MRI finding highly suggestive of PA is sphenoid sinus mucosal thickening. This is probably related to local venous congestion during the acute phase of bleeding and tends to resolve with time [102,103,104].

If MRI is contraindicated or not available, computed tomography (CT) may be used. CT effectively visualizes expansile pituitary lesions resulting in sella turcica enlargement. A CT scan detects the presence of a sellar mass in more than 80% of cases, but only diagnoses less than 30% of PAs [105,106]. Due to the hemorrhagic component of most PAs, CT shows patchy or confluent hyperdensities within a lesion [99].

Visual acuity, visual field, and ocular motility should be assessed periodically at the patient’s bedside during the first 48 h and, if possible, an instrumental examination (Humphrey analyzer or Goldman’s perimeter) should be performed [107,108].

Finally, pituitary hormones should be measured to detect possible pituitary hormone deficiencies as well as endocrine conditions of hormone hypersecretion. Blood samples should be taken urgently for the measurement of electrolytes, renal function, liver function, coagulation, blood count, and pituitary function (cortisol, prolactin, free thyroxine, TSH, IGF-1, GH, LH, FSH, and estradiol in women of childbearing age or testosterone in men) [108].

It is important to differentiate PA from other entities that may cause similar symptoms, such as internal carotid artery aneurysmal rupture [109], bacterial meningitis [80], subarachnoid hemorrhage [110], and adult optic neuritis [111].

## 8. Management

The ideal therapeutic approach of PA is not clearly defined and remains controversial. While some authors favor early transsphenoidal surgical decompression in all patients, others recommend a conservative approach in selected patients [55,112]. It is important to note that the extent of hemorrhage/ischemia, the symptomatology at presentation, and the clinical course of PA can vary considerably from patient to patient. Therefore, symptoms may vary from a mild headache with mild visual disturbances occurring over several days to a severe headache of abrupt onset with severe visual disturbances and even decreased level of consciousness. However, most patients have symptoms between these two extremes. Patients without neuro-ophthalmologic symptoms or with mild and stable symptoms and/or signs may be managed conservatively under close supervision. On the other hand, patients with worsening visual acuity, level of consciousness, or visual field defects should undergo urgent MRI to plan surgical decompression. In any case, because PA is a medical emergency, treatment must be individualized and tailored to each patient after a thorough evaluation by a multidisciplinary team, including a neuroradiologist, an endocrinologist, an ophthalmologist, and a neurosurgeon with expertise in pituitary pathology [55,108,112].

### 8.1. Conservative Approach

Patients without neuro-ophthalmologic symptoms or with mild and stable symptoms and/or signs may be managed conservatively with close monitoring of neurologic symptoms, visual acuity and visual field loss, renal function, and electrolytes. Similarly, patients with subacute PA or those with prolactinoma can be managed conservatively, in the latter case with the addition of dopamine agonists.

**Figure 4:**
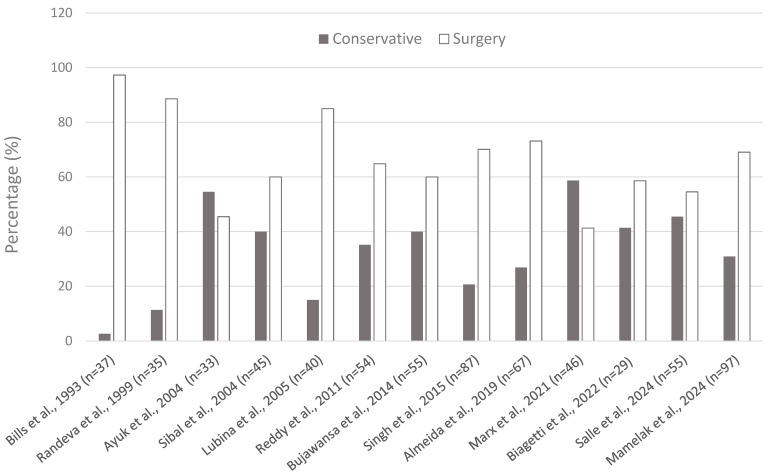
Distribution of conservative and surgical approaches in different series of patients with pituitary apoplexy [7,10,36,52,105,113,114,115,116,117,118,119,120].

Conservative treatment should be initiated in all patients with PA, although it is usually used as the sole treatment in only about one-third of patients. An analysis of 708 patients with PA from several series (each with more than 30 patients) published in the last 30 years showed that 30.6% of the patients received exclusively conservative treatment, while the rest (69.4%) were additionally treated with surgery (Figure 4).

The goals of pharmacologic treatment are to correct corticosteroid deficiency, prevent associated complications, and reduce morbi-mortality [34,55,59,108,121].

The conservative approach focuses primarily on the administration of high-dose corticosteroids, hemodynamic stabilization, administration of hormone replacement therapy for hormonal deficiencies, medical management of functioning PitNETs (such as prolactinomas, acromegaly, and Cushing’s disease), and specific treatment of any other underlying causes responsible for PA.

The main reason for the use of high doses of glucocorticoids is their anti-inflammatory and antiedematous effects. They are also used as replacement therapy because a high percentage (70%) of patients with PA develop central adrenal insufficiency, which is the hormonal deficiency most often associated with mortality in these patients. Hypocortisolemia is a predisposing factor for hemodynamic instability because it is associated with a decreased vascular response to catecholamines. It also increases the release of arginine vasopressin, which decreases the excretion of free water and facilitates the development of hyponatremia [59].

Hydrocortisone 100–200 mg intravenous (iv) bolus followed by 2–4 mg every hour by continuous iv infusion or 100 mg intramuscular (im) bolus followed by 50–100 mg every six hours by im injection is commonly used. Dexamethasone (2 to 16 mg per day orally or iv in two or three doses) may be useful initially to reduce edema if early surgical decompression is not performed. In some cases, it may be necessary to add mannitol [59,108,122].

During the course of conservative treatment, evaluation of neurologic symptoms is essential, initially on an hourly basis, which may be increased to 4–6 hourly intervals if there is improvement. Visual acuity and visual field should also be monitored daily until improvement is noted, and fluid and electrolyte levels should be monitored daily or more frequently if necessary. In the follow-up, MRI should be scheduled urgently if a decrease in the level of consciousness, a deterioration in visual acuity, or a worsening of the visual field is observed.

After initial improvement of the acute episode, it is recommended to rapidly taper the glucocorticoid dose to 20 to 30 mg of hydrocortisone per day orally divided into three doses.

### 8.2. Surgical Approach

**Figure 5:**
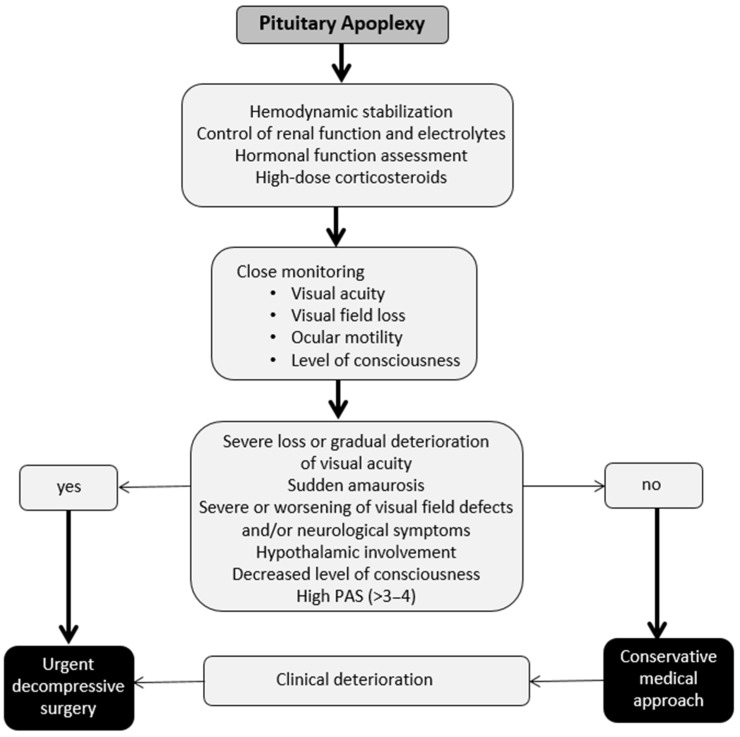
Suggested therapeutic management of pituitary apoplexy due to pituitary hemorrhage associated with compressive symptoms due to compression of adjacent structures.

In most reported series [7,10,36,52,105,113,114,115,116,117,118,119,120], urgent surgical intervention is recommended in situations involving decreased level of consciousness, hypothalamic involvement, sudden amaurosis, or loss of visual acuity. Similarly, intervention is suggested in cases of gradual deterioration of visual acuity, worsening of visual field defects, and/or neurological symptoms (Figure 5).

If surgical decompression is chosen, the transsphenoidal approach is now preferred because it allows adequate decompression of the visual tracts and adjacent structures in contact with the PA tissue lesion, with lower morbidity and mortality [34,55,108,121]. Although both microscopic and endoscopic surgery are viable options, the endoscopic route is currently more commonly used, and sometimes it may be necessary to resort to open surgery via craniotomy [119]. Surgery should be always performed by a neurosurgeon with experience in pituitary pathology.

Regarding the timing of surgery, although some studies have suggested that early surgery (within the first 7 days) could be associated with a more favorable prognosis [52,123], this has not been confirmed by a recently published prospective, multicenter, observational study [119]. This study showed that early surgery (≤2, ≤3, or ≤4 days) did not have a significant impact on outcomes 3 months after the onset of PA.

Decompressive surgery is justified by the improvement in visual acuity, campimetric defects, ophthalmoplegia, and hormonal deficits observed in most patients after surgery [124,125,126,127].

Close monitoring is essential after decompressive surgery, especially in the first 24–48 h. Water–electrolyte balance, plasma and urine osmolality, the neurological status of the patient, and the evolution of visual deficits, including visual acuity, eye movements, and confrontation campimetry, should be closely monitored.

Complications associated with PA surgery that require readmission within the first 30 days after hospital discharge are uncommon (~6%). These include CSF leak, hyponatremia, and epistaxis [119]. Other complications that may occur postoperatively are diabetes insipidus, infection, hypopituitarism, and neurological or visual impairment [124,125,126,128].

### 8.3. Conservative Treatment vs. Surgery

PA has traditionally been considered a neurosurgical emergency, however, some retrospective and prospective studies have shown similar results between medically treated and surgically intervened patients (Table 1).

A recently published systematic review and meta-analysis on surgical versus conservative management in the treatment of PA showed that the recovery rates of visual field, endocrine function, and ophthalmoplegia were 76%, 23%, and 81%, respectively, for surgery and 79%, 23%, and 90%, respectively, for conservative treatment [129]. However, it should be taken into account that the groups studied are not usually homogeneous at baseline because the proportion of patients with ophthalmological signs at the time of PA presentation is significantly higher in the surgically treated group than in the conservatively treated group [36,120].

The main controversy in treating PA lies in determining when neurosurgical decompression is indicated and when to perform such decompression. In order to be able to better compare different therapeutic options (conservative approach vs. immediate neurosurgical intervention) and help make a therapeutic decision the UK guidelines of the Pituitary Apoplexy Guidelines Development Group for the management of PA proposed in 2011 the Pituitary Apoplexy Score (PAS) based on level of consciousness (Glasgow Coma Scale (GCS) score) and ophthalmological data (visual acuity, visual field defects, and ocular paresis) [59] (Table 2).

Several studies have retrospectively evaluated the PAS in relation to the type of treatment used in PA [36,116,117,118] (Table 3). PAS value was significantly lower in the group of patients treated conservatively compared to those who underwent surgery. In the conservative group, PAS ranged between 1 and 2, while in the surgical group, it ranged between 3 and 4. In addition, PAS was the only variable that was associated with surgical treatment in the multivariate analysis in one of the above-mentioned studies [36]. In general, it can be concluded that the prediction of adequate treatment affects patients with a PAS around 3–4.

Some authors [118] have suggested the inclusion of headache as part of the PAS, as it may be a factor indicating the need for surgery in certain situations, especially in the case of headache refractory to analgesic treatment. However, the perception of headache intensity is subjective and could lead to selection bias. Within the PAS, the score for GCS and for cranial nerve palsies has not shown statistically significant differences between the treatment groups in the different studies evaluated [36,116,117,118]. On the other hand, since most vigilance disorders are associated with corticotropin deficiency and are reversible with glucocorticoid hormone replacement therapy, it has been suggested that PAS should always be evaluated after correct hormonal supplementation [36].

It has been criticized that PAS does not consider pituitary hormone function. Although no differences have been found in the effects on hormonal function between the types of treatment at the time of PA, some authors have suggested that serum prolactin concentrations could serve as a predictor of the capacity for recovery of pituitary function in the medium and long term [36,130]. Low prolactin levels could indicate greater pituitary parenchymal damage and be associated with a lower capacity for recovery of hormonal function after PA [36]. This raises concerns about the usefulness of surgery in hypoprolactinemic patients when the primary goal is to restore pituitary function.

Recently, a prospective, international, multicenter study compared the presentation and outcomes of patients with PA undergoing surgical versus non-surgical management [119]. In this study 97 patients were analyzed (30 received medical treatment and 67 underwent surgery). The treatment decision for each patient was made on an individual basis based on investigators’ best judgment and treatment preferences. Baseline clinical characteristics, including demographics, presenting symptoms, hormonal deficiencies, and imaging study findings, were comparable in both groups. Patients with PAS equal to or greater than 3 were more likely to undergo surgery than patients with PAS 0 to 2 (*p* = 0.03). At presentation, only visual field defects and optic nerve compression on MRI showed significant differences between the groups, with the surgical group presenting the most severe visual field defects. Hormonal, visual, and oculomotor outcomes were similar at 3 and 6 months. Stratification by visual field severity showed no significant differences in any outcome at 3 months. In addition, no association was found between the timing of surgery and any of the outcomes. Importantly, apoplectic tumor volume decreased significantly at 2–3 months even in conservatively treated patients, suggesting that surgery is not always necessary to reduce mass effect. Consequently, this study failed to demonstrate an association between surgery and better outcomes in PA [119]. However, when interpreting these results, it is important to keep in mind the selection bias, as patients treated with surgery tend to be those with severe symptoms at the onset of PA or who have worse outcomes with conservative treatment.

Another proposed classification system for PA which has implications for management, outcomes, and categorization for future studies is the Pituitary Apoplexy Grading System (PAGS) which divides patients into five grades of increasing clinical severity based on the most common presenting symptoms: Grade 1: asymptomatic patients (subclinical apoplexy); Grade 2: symptoms attributable exclusively to hypopituitarism; Grade 3: headache (acute-onset new or acute-on-chronic); Grade 4: ocular paresis (cavernous sinus cranial nerves); and Grade 5: visual deficits or a low GCS score that precludes visual assessment [131]. Patients with low grades (Grades 1 to 3) had a more frequent complete recovery from endocrine deficiency than patients with higher grades (Grades 4 to 5). In addition, there was a trend toward earlier surgical treatment in patients with higher grades, with good overall outcomes in patients with all grades.

## 9. Short- and Long-Term Surveillance

After an acute episode of PA, close follow-up in the short, medium, and long term is recommended for all patients. This is essential to assess the patient’s progress, detect possible complications, adjust treatment if necessary, and provide appropriate support for rehabilitation. This follow-up can include imaging studies, neuro-ophthalmological and endocrine assessments, as well as emotional support and physical rehabilitation according to the individual needs of each patient, allowing for comprehensive and personalized management of each case and optimizing long-term outcomes.

During the course of conservative treatment, regular assessments of neuro-ophthalmological symptoms such as visual acuity and visual fields are necessary. Ideally, these assessments should be performed hourly initially, considering increasing intervals to 4–6 h if significant improvement is observed. Fluid and electrolyte balance be monitored on a daily basis, or more frequently as required. In the event of obvious clinical deterioration, particularly in the form of decreased visual acuity or field and/or decreased level of consciousness, an MRI should be performed urgently and urgent surgical decompression should be considered [55,59,108,122].

In patients who have undergone decompressive surgery, care in the immediate postoperative period is similar to that after surgery for PitNET. Possible complications in this period include diabetes insipidus, central adrenal insufficiency, visual loss, CSF fistula, and meningitis. It is therefore essential to monitor fluid balance and assess adrenal and thyroid function postoperatively. Cortisol measurement at 8 a.m. is the recommended test for the initial assessment of the newly operated patient in the absence of steroid therapy. Similarly, thyroid function (TSH and free thyroxine) should be assessed from the third or fourth postoperative day. If normal, it should be reassessed after 6–8 weeks. After surgery, it is common to see improvement in visual acuity, campimetric defects, and oculomotor palsy in most patients. This improvement is usually seen in the immediate postoperative period and often continues for a few weeks after the operation [59,108].

Following an episode of PA, partial or complete hypopituitarism often develops in the following days or months, which may be transient or permanent. Approximately 80% of patients require some form of hormone replacement therapy and around 50% of patients experience partial or complete recovery of pituitary function. Complete recovery of pituitary function occurs in only 5–37% of patients [55].

Visual symptoms may improve with follow-up, although recovery is less likely in patients with unilateral or bilateral vision loss. Diplopia tends to improve first, while visual field loss improves more slowly. Retinal optical coherence tomography may help to determine the prognosis of visual field loss [132,133].

Although spontaneous cure of functioning and non-functioning pituitary tumors after PA has been described [76,77], recurrence of PA, as well as tumor growth or recurrence [7], has been observed similarly in both conservatively and surgically managed patients [120]. Therefore, prolonged follow-up of all patients is essential to detect any recurrence regardless of the therapeutic modality used.

## 10. Conclusions

PA is a rare but potentially serious condition that is difficult to diagnose due to its variable and often non-specific clinical presentation. MRI is the diagnostic tool of choice because it can show typical features such as areas of pituitary hemorrhagic necrosis. Initial medical management of acute AP is aimed at control of symptoms (such as headache, nausea, vomiting, etc.), hemodynamic stabilization, monitoring of hormonal and renal function, electrolytes, and administration of high-dose corticosteroids. In selected cases, such as asymptomatic patients or those with mild symptoms, a conservative approach with symptomatic management and close follow-up may be considered instead of immediate surgical intervention. However, in cases of severe symptoms or compression of adjacent structures, transsphenoidal surgery may be required to decompress the pituitary gland and relieve symptoms. Surgery may also be required in cases of massive hemorrhage or severe neurological symptoms. There are PA score systems that can help in making therapeutic decisions. After initial treatment, close follow-up is required to assess hormonal function and possible long-term complications, such as new hormone deficiencies and the possibility of recurrence of PitNETs from postoperative tumor remnants. Given the complex nature and consequences of PA, optimal management requires close collaboration among multidisciplinary team members including emergency physicians, endocrinologists, neurosurgeons, neuro-ophthalmologists, neuroradiologists, and intensivists. Given the possibility of short- and long-term sequelae, recovery of pituitary function, recurrence of PA, and growth of underlying PiTNETs, short- and long-term follow-up is recommended.

## Figures and Tables

**Table 1:** Clinical features, therapeutic management, and outcomes of published series of pituitary apoplexy in the last ten years comparing surgery vs. conservative management.

Author et al., Year (Ref.)	Type of Study	Number of Patients	Sex (M/F)	Surgery *n* (%)	Conservative Management *n* (%)	Outcomes (Surgery vs. Conservative Management)
Bujawansa et al., 2014 [117]	Retrospective	55	35/20	33 (60)	22 (40)	No statistically significant difference in the rates of complete or near-complete resolution of visual field deficits and cranial nerve palsies and endocrine outcomes between those treated conservatively and those who underwent surgical decompression
Singh et al., 2015 [114]	Retrospective	87	57/30	69 (77.3)	18 (20.7)	No statistically significant differences in any of the outcome measures across the treatment groups
Almeida et al., 2019 [120]	Retrospective	67		49 (73.1)	18 (26.9)	No significant difference between conservative and surgical treatment for visual field recovery, cranial nerve recovery and hypopituitarism, or tumor recurrence was observed
Marx et al., 2021 [118]	Retrospective	46	29/17	19 (41.3)	27 (58.7)	Improvements in visual acuity, cranial nerve palsy, and visual field were similar, and the endocrine prognosis remained poor after pituitary apoplexy, regardless of the therapeutic approach Higher number of hormonal deficits in the surgery group at 1 year
Biagetti et al., 2022 [115]	Retrospective	29	15/14	17 (58.6)	12 (41.4)	Non-statistical differences in outcomes were found between patients who underwent surgery or were conservatively treated
Salle et al., 2024 [36]	Retrospective	55	36/19	30 (54.5)	25 (45.4)	No significant differences in ophthalmological parameters and endocrine function at both the 3-month and 1-year follow-up All oculomotor disturbances regressed and normalized by the 3-month follow-up
Mamelak et al., 2024 [119]	Prospective, multicenter, observational	97	68/29	67 (69.0)	30 (31.0)	No differences in hormonal recovery, visual field, and oculomotor palsy outcomes at 3 months

M, males; F, females.

**Table 2:** Pituitary apoplexy score (PAS) [59].

Variable	Points
Level of consciousness	
GCS 15	0
GCS < 8–14	2
GCS < 8	4
Visual acuity	
Normal * 6/6	0
Reduced—unilateral	1
Reduced—bilateral	2
Visual field defects	
Normal	0
Unilateral defect	1
Bilateral defect	2
Ocular paresis	
Absent	0
Present—unilateral	1
Present—bilateral	2

GCS: Glasgow Coma Scale. * No change from premorbid visual acuity.

**Table 3:** Pituitary apoplexy score (PAS) value according to the type of therapy used in pituitary apoplexy in retrospective studies.

	Conservative Group	Surgery Group	*p*-Value
Reddy et al., 2011 [116] (*n* = 54)	*n* = 19	*n* = 35	
	1.4 (range, 0–4)	3.5 (range, 1–9)	NA
Bujawansa et al., 2014 [117] (*n* = 46)	(*n* = 27)	(*n* = 19)	
	1.8 (range, 0–5)	3.8 (range,1–6)	<0.01
Marx et al., 2021 [118] (*n* = 46)	(*n* = 27)	(*n* = 19)	
	1.1 (range, 0–4)	3.0 (range, 1–6)	0.0002
Salle et al., 2023 [36] (*n* = 55)	(*n* = 25)	(*n* = 30)	
	1.7 ±1.7	3.9 ± 1.7	<0.0001

NA: not available.
